# Primary CD5-negative intrathyroidal thymic carcinoma with neuroendocrine differentiation and lymph node metastasis acquiring CD5 expression:case report

**DOI:** 10.1016/j.bjorl.2026.101834

**Published:** 2026-06-16

**Authors:** Lijun Wen, Li Zhou, Min Wang, Guangzhen Ma, Wei Hong

**Affiliations:** a The Second People's Hospital of Liaocheng, Department of Pathology, Linqing City, Shandong Province, China; b The Second People's Hospital of Liaocheng, Department of Oncology, Linqing City, Shandong Province, China

**Keywords:** Immunohistochemistry, Clonal evolution, Diagnostic challenge, Rare tumors, Papillary thyroid carcinoma

## Introduction

Intrathyroidal Thymic Carcinoma (ITTC), or Carcinoma Showing Thymus-Like Differentiation (CASTLE), accounts for less than 0.15% of all thyroid malignancies.^1^ It is thought to arise from ectopic thymic tissue or branchial pouch remnants. ITTC typically follows an indolent clinical course and is often diagnosed at a locally advanced stage as an asymptomatic neck mass. Preoperative cytological diagnosis is challenging, and definitive diagnosis relies on postoperative histopathology and Immunohistochemistry (IHC). The classic immunoprofile of ITTC includes consistent expression of CD5 and CD117, which are considered key diagnostic markers.^2^ CD5 is a transmembrane protein expressed on T-cells and a subset of B-cells. Its reported sensitivity in ITTC ranges from 82% to 100%.^3^^,^^4^ However, emerging evidence suggests immunophenotypic heterogeneity, particularly in advanced cases. We present a diagnostically challenging case of primary CD5-negative ITTC with neuroendocrine differentiation and concurrent Papillary Thyroid Carcinoma (PTC), further complicated by heterogeneous CD5 expression in the recurrent lesion, suggesting clonal evolution.

## Case report

A 32-year-old married Chinese woman presented in 2019 with a 7-day history of hoarseness and aspiration while drinking. Physical examination revealed a 3 cm firm, mobile mass in the left thyroid lobe. Ultrasound identified a 4.1 cm lesion in the left lobe and a 0.2 cm nodule in the right lobe. Fiberoptic laryngoscopy revealed left vocal cord paralysis. Contrast-enhanced Computed Tomography (CT) confirmed a 3.2 cm hypodense nodule in the left lobe. The patient underwent total thyroidectomy, central neck dissection and left cervical lymphadenectomy. Upon reviewing the histopathology slides of the primary tumour, the following features were observed: an infiltrative growth pattern, lobulated nests separated by fibrous septa, and lymphocytic stroma. Immunohistochemistry showed positivity for CD117, p63, and p40, and negativity for CD5, TTF-1, and thyroglobulin. Based on these findings, the diagnosis was intrathyroidal thymic carcinoma. A month post-surgery, she received radioactive iodine ablation. Six years later, routine follow-up detected enlarged left cervical lymph nodes. The cellular morphology is consistent with metastatic carcinoma. Given the patient’s clinical history, metastasis of ITTC remains a plausible diagnostic consideration. Pathology after lymph node excision indicated metastasis from thymic carcinoma originating in the thyroid. The patient sought external consultation and commenced chemotherapy (Table 1). At the most recent evaluation, she remained stable, performed normal daily activities, and continued regular follow-up as advised.

**Table 1:** Clinical timeline.

Time	Diagnostic findings	Treatment	Pathology
July 2019	Ultrasound: 4.1 cm mass in the left thyroid gland; 0.2 cm nodule in the right gland	Total thyroidectomy with central neck dissection and left cervical lymphadenectomy, along with exploration of the recurrent laryngeal nerve	Intrathyroidal Thymic Carcinoma and Papillary Thyroid Carcinoma
	CT: 3.2 cm hypodense nodule		
	Fiberoptic laryngoscopy left vocal cord paralysis		
August 2019	NA	Radioactive iodine ablation therapy	NA
March 2025	Physical examination: Enlarged left cervical lymph nodes	Fine-needle aspiration biopsy	Metastatic thymic carcinoma (thyroid origin)
	Ultrasound:	Surgical excision of the lymph nodes	Thymic carcinoma metastasis originating from the thyroid
August 2025		commenced chemotherapy in an external institution	Thymic carcinoma metastasis

**Fig. 1:**
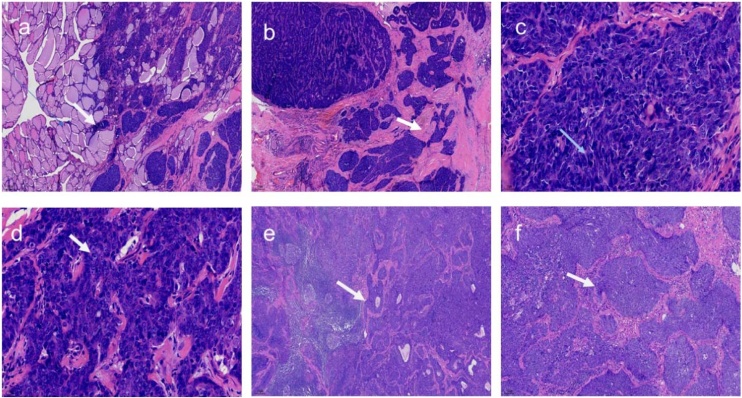
(a) Tumor lacks a well-defined capsule and demonstrates a permeative infiltration pattern within the thyroid parenchyma (white arrow) H&E ×40. (b) Neoplastic cells are arranged in variably sized nests and interconnected trabeculae separated by prominent fibrous septa (white arrow) that create a distinct lobular architecture H&E ×40. (c‒d) Tumor cells exhibit polygonal to short spindle-shaped morphology (blue arrow) with poorly defined cytoplasmic borders. The nuclei display round to oval contours, with focal nuclear clearing and prominent small nucleoli in some cells (white arrow) H&E ×400. (e‒f) Lymph node metastasis recapitulated the primary tumor morphology (white arrow) H&E ×100.

The resected left thyroid lobe contained a 4 cm gray-white nodular lesion with friable consistency and infiltrative borders. The right lobe contained a 0.2 cm firm, gray-red nodule. Histologically, the primary tumor lacked a well-defined capsule and showed infiltrative growth into adjacent skeletal muscle and adipose tissue (Fig. 1a). The tumor cells were arranged in nests and interconnected trabeculae separated by prominent fibrous septa, creating a lobular architecture (Fig. 1b). Lymphocytic infiltrates were present in the stroma, and cells exhibited polygonal to short spindle shapes with poorly defined cytoplasmic borders (Fig. 1c). Nuclei were round to oval, with focal clearing and small prominent nucleoli in some cells, while others showed finely dispersed chromatin and inconspicuous nucleoli (Fig. 1d). Small necrotic foci occurred within cell nests, and rare mitotic figures were observed. The metastatic lymph node lesions recapitulated the morphology of the primary tumor (Fig. 1e‒f).

**Fig. 2:**
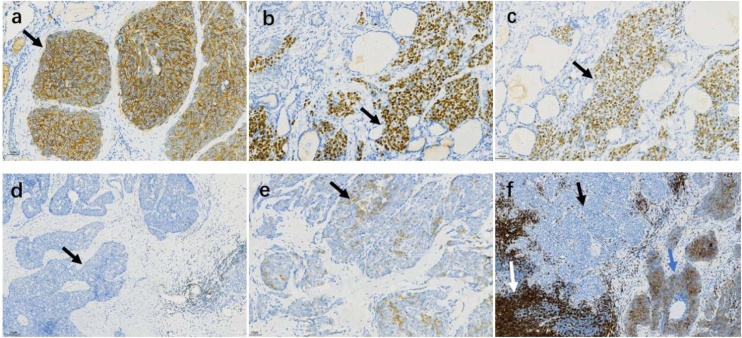
Immunohistochemistry results of tumor cells showing positivity for CD117 (a), P40 (b), and P63 (c) (black arrow). The immunohistochemistry results of tumor cells were negative for CD5 (d) (black arrow), and positive lymphocytes in the stromal region at the lower right corner may serve as a reliable internal control. Few tumor cells expressed synaptophysin (Syn) (e) (black arrow). IHC × 200 Partial expression of CD5 was observed (f). Positive lymphocyte expression in the lower left corner served as an internal control (white arrow). In contrast, tumor cells in the lower right corner presented positive expression (blue arrow), whereas those in the upper middle region presented negative expression (black arrow) IHC × 100.

Immunohistochemically, the primary tumor cells were positive for CD117, P40, and P63 (Fig. 2a–c), but negative for CD5 (Fig. 2d), TTF-1, calcitonin, PAX-8, thyroglobulin, and CD56. Focal expression was observed for Synaptophysin (Syn) and Chromogranin A (CgA) (Fig. 2e). The Ki-67 proliferation index was 30%. In the lymph node metastases resected six years later, the tumor cells were positive for CD117, P40, and CK19, and showed partial positivity for CD5 (Fig. 2f) and p16. INSM1 and synaptophysin showed focal or scattered positivity, while TTF-1 was negative. Two consecutive EBER in situ hybridization tests were negative. Based on the integrated morphological and immunohistochemical findings, the final diagnosis was CD5-negative ITTC with neuroendocrine differentiation, concurrent with PTC.

## Discussion

ITTC is a rare malignant first reported in 1985. The WHO classified it in 2004 as “CASTLE” and reclassified it in 2017 as ITTC. While typically primary to the thyroid, it can also occur in the salivary glands and neck soft tissue. Neuroendocrine differentiation, evidenced by Syn and CgA expression, is present in 22%–40% of cases. Diagnosis relies heavily on IHC to distinguish it from squamous cell carcinoma, anaplastic thyroid carcinoma, and metastatic tumors.^2^ The classic immunoprofile of CD5 and CD117 positivity is a cornerstone of diagnosis. The absence of CD5 expression, as in our primary tumor, poses a significant diagnostic challenge. However, the negative staining for thyroglobulin, TTF-1, and PAX-8 effectively ruled out a follicular epithelial-derived tumor, while the absence of calcitonin excluded a medullary carcinoma. The co-expression of CD117, p63, and p40 strongly supported a diagnosis of ITTC or primary thymic squamous cell carcinoma. The lack of a mediastinal mass made the latter highly unlikely. The histomorphology ‒ infiltrative growth, lobulated nests, fibrous stroma, and lymphoplasmacytic infiltrates ‒ was also characteristic of ITTC.

Our findings confirm and extend recent insights into ITTC heterogeneity. While CD5 was long considered a near-obligate marker, newer studies report exceptions. Gao et al. observed variable expression of in some ITTCs.^2^ Łukasiewicz et al. reported a case of ITTC with CD117 and p40 expression and only partial CD5 expression,^4^ and a case of completely CD5-negative ectopic thymic carcinoma of the parotid gland have been documented.^5^ Currently, the number of relevant cases remains limited. Notably, we document the first instance of CD5 acquisition over time. Although Sasaki et al. affirmed CD5’s utility in salivary gland ITTC, they did not address temporal immunophenotypic change.^3^ In contrast, therapy-driven phenotypic shifts (e.g., CD20 loss after rituximab in lymphoma; antigen loss in lung cancer) are well established. Our case positions ITTC within this paradigm of tumor adaptability ‒ supporting its recognition as a dynamic neoplasm, not a static curiosity.

The most intriguing aspect of our case is the discordant CD5 expression between the primary and metastatic tumors. Technical artifacts are an unlikely explanation, as internal controls (lymphocytes) showed robust CD5 staining. CD5 expression in metastatic lesions provides strong evidence of immunophenotypic evolution in ITTC, reflecting the tumor’s biological plasticity. This shift may arise through two non-mutually exclusive mechanisms: (1) Radioactive iodine ablation of concurrent PTC may have enriched a pre-existing, CD5-positive subclone from the primary tumor ‒ too rare for detection by routine IHC; (2) Metastasis-associated processes (e.g., EMT and nodal microenvironment adaptation) may reactivate CD5 via epigenetic modifications. Our case emphatically demonstrates that CD5 negativity does not exclude ITTC when characteristic histomorphology and a supportive IHC profile (CD117/p63 positivity, thyroglobulin/TTF-1 negativity) are present.

## Conclusion

This case expands the clinicopathological spectrum of ITTC by illustrating primary CD5 negativity, neuroendocrine differentiation, and concurrent PTC. Crucially, it provides the first evidence of clonal evolution manifesting as acquired CD5 expression in a metastatic lesion. Based on the foregoing analysis, we derive the following two conclusions. First, CD5 negativity does not rule out ITTC when morphology and CD117+/p63+ staining is consistent. Second, immunophenotypic evolution can occur. Repeat biopsy and IHC re-evaluation are advised for recurrent or metastatic disease.

## ORCID ID

Lijun Wen: 0000-0001-8713-499X

Li Zhou: 0009-0004-8028-6463

Min Wang: 0000-0002-6923-7466

Guangzhen Ma: 0000-0002-2242-511X

Wei Hong: 0009-0002-4412-2088

## Authors’ contributions

All the authors contributed to the study conception and design. Material preparation, data collection and analysis were performed by Li Zhou, Min Wang and Guangzhen Ma. The first draft of the manuscript was written by Lijun Wen and edited by Wei Hong. All the authors read and approved the final manuscript.

## Informed consent

The patients voluntarily agreed to the publication of their anonymized medical data, including clinical details and imaging and pathology findings.

## Funding

This research did not receive any specific grant from funding agencies in the public, commercial, or not-for-profit sectors.

## Data availability statement

The datasets presented in this article are not readily available because privacy needs to be protected. The requests to access the datasets should be directed to the first author at wenlijun0707@163.com.

## Declaration of competing interest

The authors declare no conflicts of interest.
